# Microfluidic Rapid Fabrication of Tunable Polyvinyl Alcohol Microspheres for Adsorption Applications

**DOI:** 10.3390/ma12223712

**Published:** 2019-11-11

**Authors:** Jianmei Wang, Xueying Wang, Pingan Zhu, Chengmin Chen, Jianchun Wang, Yan Li, Liqiu Wang

**Affiliations:** 1 Energy Research Institute, Qilu University of Technology (Shandong Academy of Sciences), Jinan 250014, China; chencm@sderi.cn (C.C.);; 2 Key Laboratory of Interfacial Reaction & Sensing Analysis in Universities of Shandong, School of Chemistry and Chemical Engineering, University of Jinan, Jinan 250022, China; chm_wangxy@ujn.edu.cn; 3 Department of Mechanical Engineering, The University of Hong Kong, Hong Kong, China; pazhu@hku.hk (P.Z.); lqwang@hku.hk (L.W.)

**Keywords:** PVA, monodisperse microspheres, adsorption, microfluidic chips

## Abstract

Monodisperse polyvinyl alcohol (PVA) microspheres have been widely used for targeted drug delivery, embolization, and templates. However, the fast and facile fabrication of PVA microspheres with uniform size and internal structure and good sphericity remains a challenge. In this study, the PVA microspheres with uniformity in the size, shape, and internal structure were rapidly fabricated, using single-emulsion droplet templates by an on-chip approach. First, we designed a polydimethylsiloxane (PDMS) microfluidic chip integrated with three functional units, used for the droplet generation, mixing of reagents, and pre-curing of PVA microspheres, respectively. Then, we precisely controlled the generation of PVA aqueous droplets, mixing of reagents, and the gelation rate for the production of high-quality microspheres by adjusting the pH value, flow rate, and the channel structure. The prepared PVA microspheres are characterized with good sphericity, uniform internal structure, and narrow size distribution. The microspheres have good adsorption capacity and recyclability for small-molecule drugs, as demonstrated by the adsorption and desorption of methylene blue (MB). The adsorption behavior is well described by the pseudo-second-order model, and intraparticle diffusion is as fast as the external film diffusion.

## 1. Introduction

Monodispersed spherical polymer microspheres play an important role in many fields due to their uniform sizes and structures, such as in controlled release of stored materials [1,2,3], fabrication of cavities or interspaces templates from microspheres [4,5], and selective adsorption or separation of substances as microreactors [6,7]. Polyvinyl alcohol (PVA) is an inexpensive, commonly used, nontoxic, biocompatible polymer, which is rich in hydroxyl groups and can be easily modified. As such, PVA is frequently used to make films, fibers, microspheres, and so on [8,9,10,11,12,13].

Many methods for preparing PVA microspheres have been reported. Yong et al. reported the electro-spray method for producing hydrogel microspheres [14]. Jia et al. reported hydrothermally prepared PVA microspheres [15]. However, polydispersity of microspheres remains the major issue with these methods. Recently, Han et al. reported a freeze-thawing method for preparing PVA microspheres [16]. Although they achieved good uniformity and sphericity, the whole preparation process is long, requiring 60 h. Young et al. have demonstrated the use of the UV polymerization method for producing PVA microspheres [17]. However, the PVA needs to be modified with methacrylate groups, and additional components, such as a UV light source are required, which increases the complexity and uncontrollability of the PVA microspheres. Minimizing the polydispersity and simplifying the production process is of great importance for improving the efficiency to expand the range of applications, using PVA microspheres.

Droplet microfluidic technology has shown great potential in the fabrication of monodisperse microspheres, due to its precise, controllable and flexible manipulation of emulsion droplets and the providing of stable reaction microenvironments [18,19]. The fabrication processes generally involve dispersing the monomers or oligomers into droplets in a continuous phase, then mixing the components in the droplet microreactors, and finally curing the materials to obtain polymer microspheres. Therefore, the droplet generation, mixing of fluids inside droplets and curing of droplets are the key processes affecting the structure and property of microspheres, and important to the rapid and stable fabrication of microspheres.

Here, we present a fast, controllable fabrication of PVA microspheres with precision by accurately controlling the generation, mixing, and curing of droplets. We designed a polydimethylsiloxane (PDMS) chip with three functional units: the droplet generation zone, mixing zone and pre-curing zone. Using the microfluidic chip, we achieved well-controlled generation of monodisperse droplets, rapid yet thorough mixing of reagents, and pre-curing of PVA droplets. Finally, the PVA microspheres with uniform size, good sphericity, and consistent internal structure were prepared, which showed good adsorption capacity and reproducibility for small-molecule drugs.

## 2. Experimental Section

### 2.1. Materials and Reagents

Polyvinyl alcohol (99% hydrolyzed, *M*_w_ = 130 kDa) was purchased from Sigma-Aldrich (Shanghai, China). Glutaraldehyde (GA, 25%–28% w/w), hydrochloric acid (HCl, 36%–38% w/w), methylene blue trihydrate, and liquid paraffin were purchased from Sinopharm (Shanghai, China). ABIL EM90 was purchased from Evonik China (Shanghai, China). Polydimethylsiloxane (PDMS) Sylgard 184 was purchased from Dow corning (Midland, USA). Trimethylchlorosilane (≥99.0%), doxorubicin hydrochloride (DOX-HCl, 98%), and Rhodamine B (>99.0%) were purchased from Aladdin water-purification system. All reagents were used as received, unless otherwise specified.

### 2.2. Experimental Method

#### 2.2.1. Design and Fabrication of Experimental Setup

**Figure 1:**
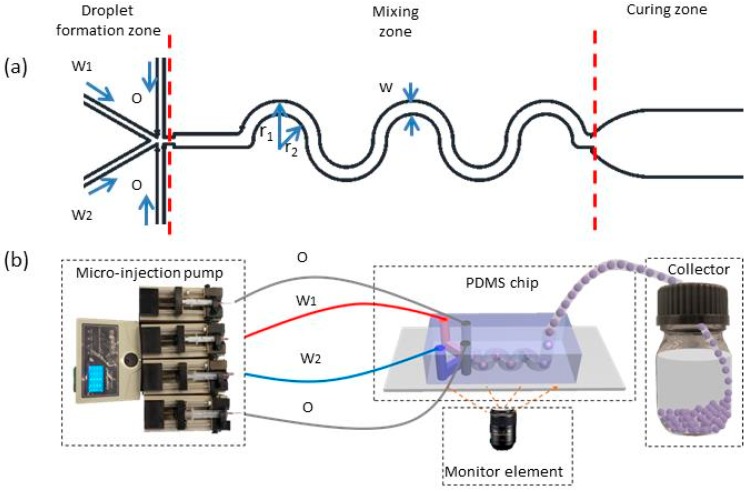
(**a**) Schematic of the PDMS chip. (**b**) The experimental setup for on-chip fabrication of monodisperse PVA microspheres.

A PDMS chip consisting of droplet generation, mixing, and pre-curing units (Figure 1a) was prepared by soft lithographic technique. A flow-focusing structure with a four-way inlet was designed for droplet generation, one of which was injected with a PVA solution (dispersed phase, inlet W_1_), one was for the mixture of GA and HCl solution (dispersed phase, inlet W_2_), and the other two were for the liquid paraffin containing 5 wt.% EM90 as the surfactant (continuous phase, inlet O). The serpentine channels were used to enhance the mixing efficiency. The PDMS chip was bonded with a glass substrate coated with a thin layer of PDMS so that all channel walls were hydrophobic to prevent the wetting by PVA. The height and width of microfluidic channels were measured by a step-measuring instrument (DektakXT, Bruker, Billerica, MA, USA). The width of the microchannels for the droplet generation, mixing, and pre-curing zone was 100, 200, and 1000 µm, respectively, and the height of all microchannels was 140 ± 5 µm unless otherwise specified.

The PDMS chip was real-time observed, using an inverted fluorescence microscope (TI-U, Nikon, Tokyo, Japan) equipped with a high-speed video camera (MIRO M310, Phantom, Wayne, NJ, USA), and was connected to syringe pumps (LSP01-2A, Baoding Longer pump, Baoding, China) and a collector, through polyethylene (PE) tubes (Figure 1b).

#### 2.2.2. Fabrication of the PVA Microspheres

The preparation of PVA microspheres contains three processes: droplet generation, fluid mixing, and PVA curing. First, PVA/GA hybrid aqueous droplets were formed at the junction of the microchannel. Then, PVA and GA were fully mixed at the mixing zone and gelated into microspheres in the pre-curing zone. Finally, the gelation microspheres were transported to the collector for deep curing to obtain PVA microspheres.

#### 2.2.3. Evaluation of Mixing Efficiency

To evaluate the mixing efficiency inside droplet, we analyzed the homogeneity of the pixel gray values of captured micrographs. A fluorescent tracer (Rhodamine B) was added into GA solution, whereas PVA solution was transparent. When mixing occurs, the concentration of the tracer changes continuously, resulting in nonuniformity in the gray value of pixels until GA and PVA are fully mixed together. The coefficient of variation (*CV*) of pixel value, reflecting the uniformity of tracer distribution, is often used to quantify the mixing efficiency. *CV* is defined as follows [20]:

(1) $$CV=\sqrt{\frac{1}{N}\sum_{i=1}^{N}{\left(\frac{G_{i}}{G}-1\right)}^{2}}\times 100\%\;$$

where *G*_i_ is the gray value of each pixel (absolute white is 255 and absolute black is 0), *G* is the mean gray value of *N* pixels, and *N* is the number of pixels selected inside a droplet in the micrograph. *CV* can vary between 1 and 0, representing no mixing and complete mixing, respectively [21]. The extent of mixing increases when droplets are transported in the serpentine channel. Therefore, we selected several featured sites in the mixing channel to calculate *CV* for the evaluation of the mixing efficiency. When the difference in *CV* between two adjacent sites was less than 0.5%, it was considered to be fully mixed, and the time required to reach this threshold is the mixing time [21].

The relationship between the mixing time (*t*, s), mixing channel length (*L*, m) and flow velocity (*u*, m/s) is below:

(2) $$t=\frac{L}{u}=\frac{n\pi (\frac{r_{1}+r_{2}}{2})\;}{u}\;$$

(3) $$u=\frac{Q_{W}{}_{{}_{1}}+Q_{W_{2}}+Q_{\mathrm{oil}}}{wh}\times 10^{-6}\;$$

where *n* is the number of half-cycle channels; *r*_1_*, r*_2_, *w*, and *h* are the inner and outer radii (Figure 1a), the width, and height of the curved channel (m); *Q*_w1_, *Q*_w2_, and *Q*_oil_ are the flow rates of PVA, the mixture of GA and HCl solution, and paraffin oil, respectively (mL/s).

#### 2.2.4. Characterization of PVA Droplets and Microspheres

The droplets were observed by optical microscopy (T-100, Nikon, Tokyo, Japan), and the microspheres were characterized by SEM (EVOMA10, Carl-Zeiss, Oberkochen, Germany). Image J and Origin software (origin 9.0) were used to analyze the size distribution of droplets and microspheres and the gray coefficient of variation. One hundred particles were analyzed in measurement. The mixing process was monitored in real time by a high-speed video camera (Phantom Micro M310, Wayne, NJ, USA) mounted on an inverted fluorescence microscope (TI-U, Nikon, Tokyo, Japan). The gelation process was monitored in real time by optical microscopy, and the degree of polymerization is expressed by the degree of phase separation. The phase separation (*σ*) is defined in Equation (4):

(4) $$\sigma =1-\frac{d_{\mathrm{gel}}}{d_{\mathrm{drop}}}\;$$

where *d*_drop_ (μm) is the size of droplets and *d*_gel_ (μm) is the gel size. The swelling ratio of the PVA microspheres was obtained by soaking the microspheres in 0.9% aqueous sodium chloride solution, for 72 h, to achieve the swelling balance. The microspheres before and after swelling were recorded with an optical microscope. The swelling ratio (*φ*) is defined as the following:

(5) $$\phi =\frac{V_{w}-V_{d}}{V_{d}}={\left(\frac{d_{w}}{d_{d}}\right)}^{3}-1$$

where *V*_w_ (μm^3^) and *d*_w_ (μm) is the volume and the diameter of the microspheres after swelling, respectively; and *V*_d_ (μm^3^) and *d*_d_ (μm) is the volume and the diameter of the microspheres before swelling, respectively.

#### 2.2.5. The Adsorption Performance of PVA Microspheres

Methylene blue (MB), a small-molecule drug doxorubicin hydrochloride (DOX-HCl), and macromolecular bovine albumin (BSA) were used as three model molecules, with different molecular weights, to explore the adsorption mechanism of PVA microspheres. A certain amount of microspheres was added to the MB solution (w/v = 19.7 μg/mL), DOX-HCl (w/v = 48 μg/mL) solution, and BSA (w/v = 23 μg/mL) solution, oscillated at 200 rpm (room temperature), followed by centrifugation. The adsorption spectra of the supernatant were then measured by an ultraviolet–visible spectrophotometer (UV–vis, TU1901, Beijing, China). The equilibrium adsorption capacity (*q*_e_, mg⋅g^−1^) and the adsorption ratio (*M*) was calculated according to Equations (6) and (7):

(6) $$q_{e}=\frac{(c_{0}-c_{e})V}{m}$$

(7) $$M=\frac{C_{0}-C_{i}}{C_{0}}\times 100\%$$

where *C*_0_ and *C*_e_ are the initial and equilibrium adsorbate concentration, respectively (mM), and *C*_i_ is the concentration of adsorbent at time *t*_i_ (mM). *V* (mL) and *m* (g) are the solution volume and weight of the adsorbent, respectively.

## 3. Results and Discussion

To achieve on-chip fabrication of PVA microspheres with uniform size, structure, and sphericity, it is essential to precisely control the processes of droplet generation, component mixing, and PVA curing. The effects of flow rate, pH value, PVA/GA ratio, microchannel size, and structure on the experimental results were studied in detail.

### 3.1. Droplet Formation

**Figure 2:**
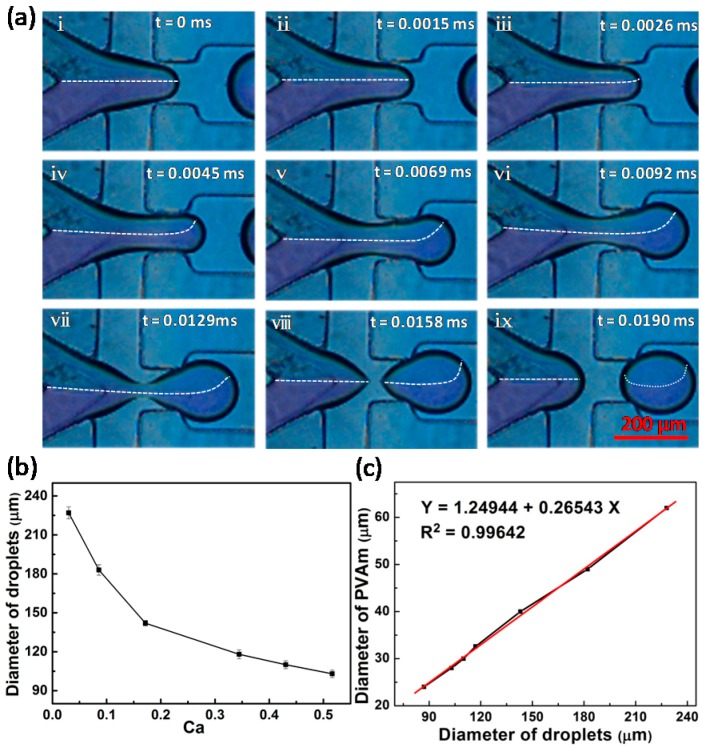
(**a**) Snapshots of droplet formation and fluid movement in droplets; (**b**) the effect of *C*a on the size of droplets at *Q*_W1_ = *Q*_W2_ = 0.3 mL/h; (**c**) the relationship between the size of PVA microspheres and that of droplets.

Figure 2 shows the formation of PVA/GA aqueous droplets dispersed in paraffin oil in the flow-focusing microfluidic device. There is a clear and stable interface between the PVA solution and GA solution at the junction (Figure 2), suggesting a constant volume ratio of the two fluids, which is critical to the fabrication of uniform microspheres. Furthermore, when the liquid finger enters the expanded channel, the liquid interface is disturbed (dashed line in Figure 2a, iv–vii) by the velocity variation inside the droplets, due to the spatial change in channel width. Therefore, back-mixing of the fluid in the liquid finger occurs. This design facilitates the mixing of the fluids in the droplets, without affecting the upstream flow of the neck, so as to keep a constant ratio of PVA to GA.

The flow rate of PVA and GA solutions obviously affect the component and size of the droplets and make it possible to adjust the size of the microspheres. Figure 2b shows the variation of droplet size with capillary number (*C*a, a dimensionless parameter representing the relative effect of viscous force versus interfacial tension), defined as the following:

(8) $$Ca=\frac{\mu v}{\eta }\;$$

where *μ* is the dynamic viscosity, *ν* is a characteristic velocity, and *η* is the interfacial tension. As *C*a increases, the droplet size decreases rapidly, and when the capillary number exceeds 0.2, the droplet size tends to approach a constant value. The relationship between the size of the solid microspheres and that of the liquid droplets is shown in Figure 2c, which exhibits a fairly good linear relationship, as a result of the uniform droplet size and constant intra-drop composition.

### 3.2. Mixing Process

**Figure 3:**
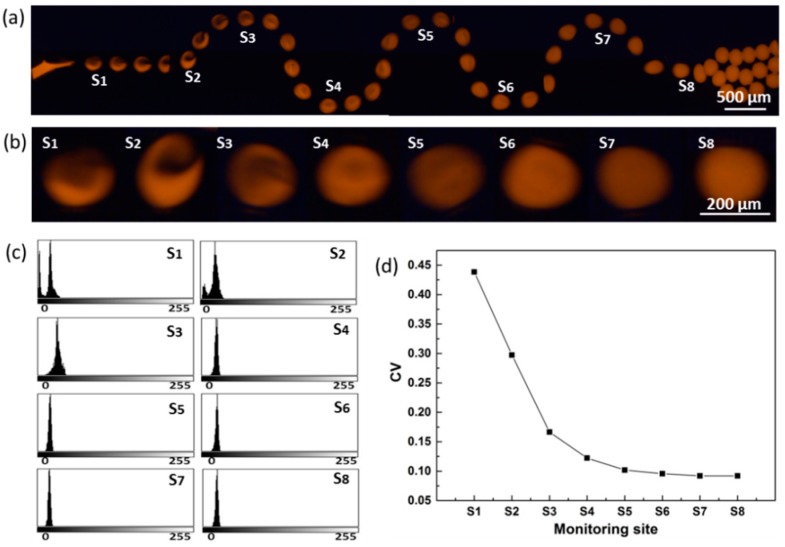
Grayscale analysis. (**a**) Fluorescent photos of two components mixed inside droplets; (**b**) magnified view of the featured site; (**c**) the grayscale distribution map of featured site; (**d**) trend of *CV* in the mixing channel.

Figure 3 shows the mixing process of the components inside the droplets and the pixel *CV* of droplets when traveling through the serpentine channel. We observed that the two components recirculate in the droplet (Figure 3a,b), and the grayscale varies from bimodal distribution to monomodal distribution, and the half-width becomes narrower from site S_1_ to S_8_ (Figure 3c), indicating that the two components tend to mix evenly. The *CV* value decreases rapidly with the increased number of half-cycle units (Figure 3d), indicating a rapid mixing of the two components. The difference of *CV* between site S_6_ and S_7_ is less than 0.5%, indicating that the two components are fully mixed.

**Figure 4:**
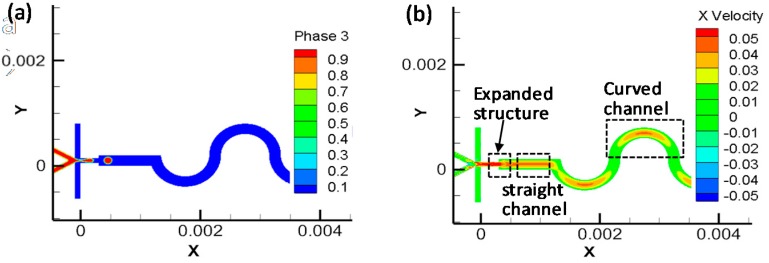
Field distribution of fluid velocity: (**a**) model diagram and (**b**) the velocity field.

The *CV*_S1_ is 0.44 (less than 1.0), illustrating that the two components start to mix due to the channel expansion during the droplet formation, which greatly shortens the mixing time and improves the mixing efficiency. We performed numerical simulations to visualize the flow field, as shown in Figure 4. When the fluids flow from the narrow channel to the wider channel downstream, the flow field is disturbed due to the flaring and releasing pressure; when the fluids enter the curved channel, the flow field is stretched and deformed, producing velocity gradients along the serpentine channel. The combination of the two structures effectively enhanced the mixing of components inside droplets.

**Figure 5:**
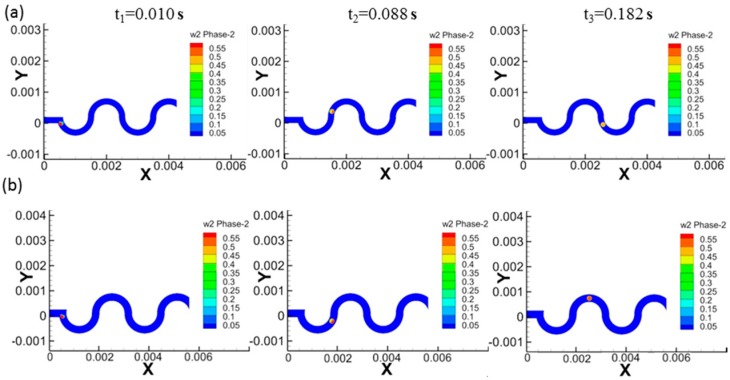
Effect of the dimension ratio of channel to droplet on the fluid-mixing efficiency. The PVA volumetric distribution inside droplet for the case of 200/200 (**a**) and 300/200 (**b**).

The effect of the dimension ratio of the channel to the droplet on the fluid-mixing efficiency is shown in Figure 5, which shows the PVA volumetric distribution (in a droplet, 0 means no PVA and 1 means an entire filling of PVA) inside the droplet for 200/200 and 300/200 (the dimension of channel to droplet with a unit of µm) cases. The volume fraction of the PVA reaches 0.5 at 0.182 s for the case of 200/200, while the fraction is 0.5–0.55 for the case of 300/200, indicating that 200/200 case has higher mixing efficiency, and showing that the droplet size has an important effect on the mixing efficiency.

**Figure 6:**
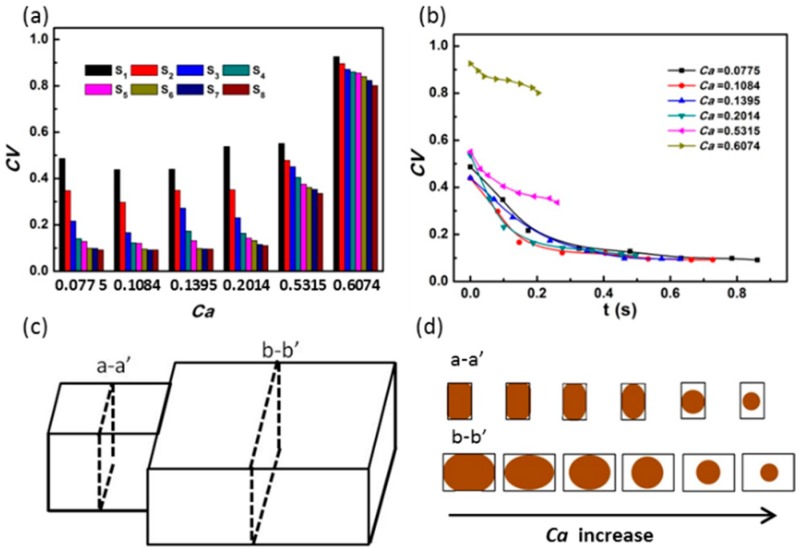
(**a**) The effect of *C*a on the coefficient of variation of pixel value; (**b**) the plot of the coefficient of variation versus mixing time; (**c**) schematic of the channel. Views a–a’ and b–b’ are the channel cross-sections; (**d**) the schematic of the state of the droplets in the channel, corresponding to the six capillary numbers in the graph (a). The height and the width for the a–a’ channel are 150 and 100 μm, respectively, and for the b–b’ channel, they are 150 and 200 μm, respectively.

The effect of capillary number *(C*a) on the mixing efficiency is shown in Figure 6. When the *C*a is below 0.5315, the first monitoring point (S_1_) shows an efficient mixing and the pixel of *CV* rapidly decreases to a stable value before reaching site S_8_; when the *C*a rises to 0.6074, the *CV* value hardly decreases within the same mixing time (Figure 6a,b). This may be due to the fact that, when *C*a is small, the droplets are squeezed in the narrow channel (Figure 6c–d, a–a’ view) and unblock the wider channel downstream (Figure 6c–d, b–b’ view), which induces a sudden release of pressure for enhancing the mixing. However, when the *C*a value is higher than 0.5315, the droplet size is too small to cause the pressure release, so that mixing of the two components inside the droplet is limited by the slow diffusion. As shown in Figure 6b, when the *C*a is smaller than 0.2014, a thorough mixing can be achieved within about 0.2 s. However, when the *C*a is higher than 0.5315, the mixing is of a low efficiency, which retards the rapid on-chip fabrication of uniform microspheres. 

### 3.3. Curing Process 

**Figure 7:**
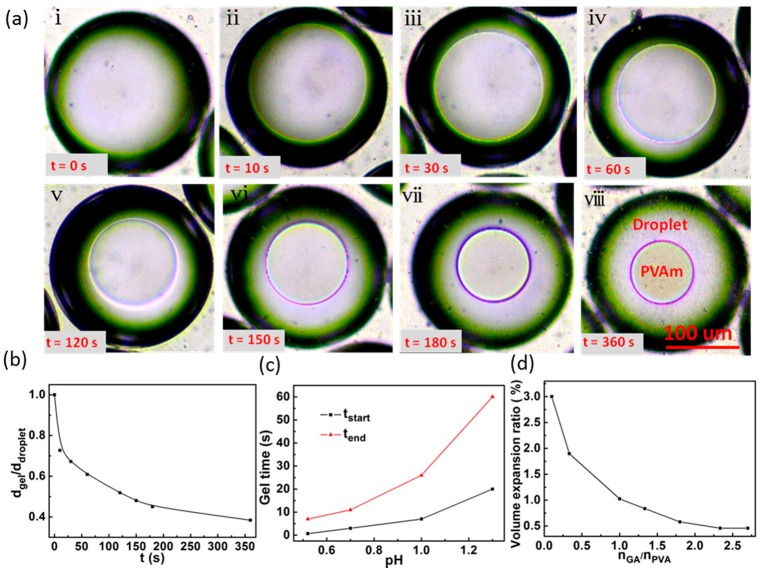
(**a**) Snapshots of the solidification of PVA and GA inside droplets; (**b**) the phase separation varies with reaction time; (**c**) the effect of pH value on the reaction rate of PVA; (**d**) the effect of GA concentration on the swelling of the PVA microspheres.

After fully mixing, the droplets entered the pre-curing zone for gel reaction, and the reaction process was shown in Figure 7a. Phase separation occurs inside the droplet to form a gel, and the gel network shrinks isotropically in the droplet, which is beneficial to the formation of spherical PVA microparticles. It is found that the degree of phase separation not only affects the sphericity of the microspheres, but also determines whether the gelation can proceed successfully. As the reaction continues, the degree of phase separation (*σ*) increases (shown in Figure 7b), so that the density of the gel microspheres becomes denser and the microspheres are robust to resist deformation, which may block the channel. To avoid such blockage, we control *σ* (see definition in Section 2.2.4) in a range of 0.2 to 0.3 for all pH values suitable for this experiment.

Figure 7c shows the effect of pH value on the reaction rate. The curing efficiency increases with the decrease of pH value. When pH = 0.52, phase separation started at 40 s and finished within 7 min, for the complete reaction, a duration that is much shorter than the time (several or more hours) consumed by using conventional methods [16,22]. Nevertheless, this method presented here requires controlling the reaction rate to prevent the formation of gel during droplet generation and mixing and to tune the internal structure of microspheres.

It was found that the amount of the cross-linking agent has a significant effect on the water-swelling of microspheres. Figure 7d shows the variation of the microspheres’ expansion ratio (*φ*, see definition in Section 2.2.4) versus GA/PVA molar ratio. When the ratio is less than 1.0, the expansion ratio increases significantly with the decrease of GA, which is useful for the medical delivery. When the ratio is greater than 1.0, the extent of swelling decreases. When GA/PVA is greater than 2.5, the expansion rate rarely changes with the increase of GA amount and reaches a stable value of about 0.45, which might be useful for the use of PVA microspheres in standard test samples or template materials.

**Figure 8:**
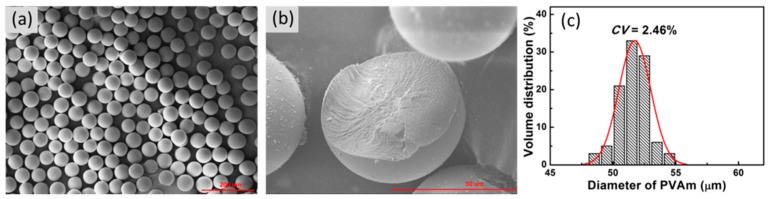
Morphology and particle size distribution of PVA microspheres. (**a**) SEM image of PVA microspheres; (**b**) SEM image of the cross section of a PVA microsphere; (**c**) size distribution of microspheres.

According to the above studies, we prepared PVA microspheres at a pH value of 0.35, the GA/PVA ratio of 0.7, and the flow rate for PVA, GA, and paraffin oil of 0.3, 0.3, and 0.4 mL/h, respectively. The chemical structure of prepared PVA microspheres was analyzed by FTIR spectrum (Vertex70, Bruker, Billerica, USA), as shown in Figure S1. FTIR result certifies that the aldehyde group crosslinks to the hydroxyl group to form PVA microspheres (PVAm). The SEM images (Figure 8) of the PVA microspheres show a good degree of sphericity, uniform internal structure, and narrow size distribution, resulting from the microfluidic-platform-enabled consistent droplet size, constant composition, well-mixing, and the stable reaction condition.

### 3.4. Adsorption Test of the PVA Microspheres

**Figure 9:**
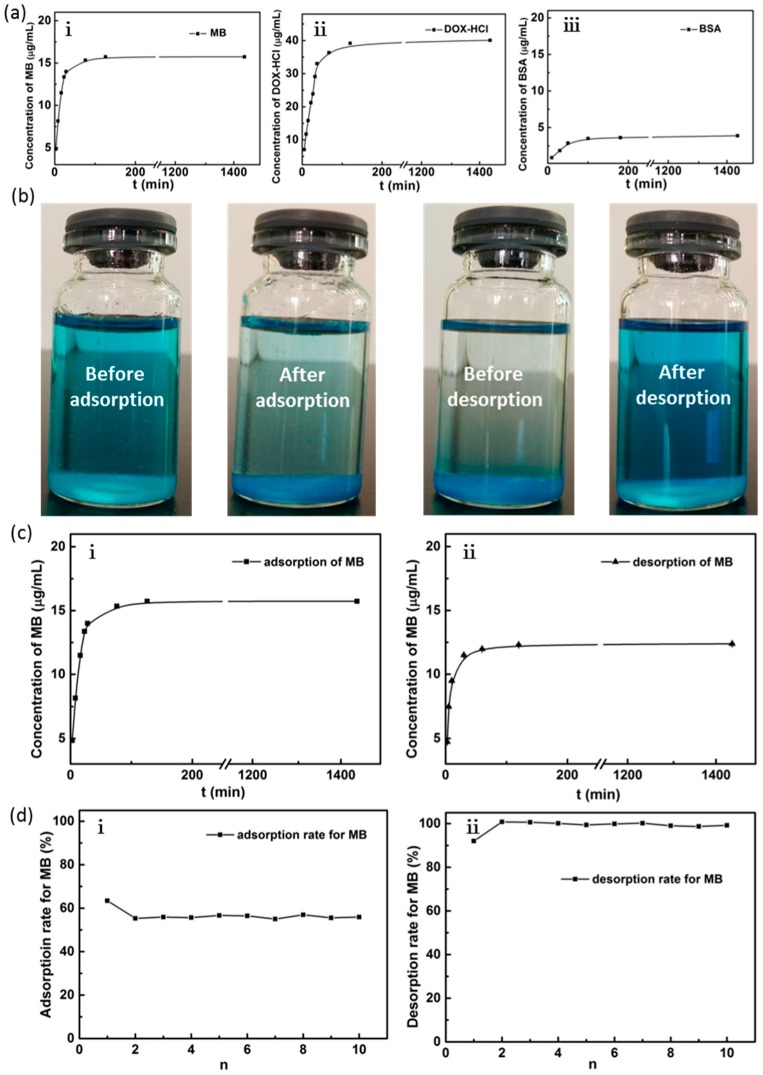
(**a**) Effect of contact time on MB; DOX-HCl and BSA adsorption onto PVA microspheres; (**b**) digital photos of adsorption–desorption MB; (**c**) effect of contact time on MB adsorption and desorption onto PVA microspheres; (**d**) the adsorption performance for 10 cycles.

The adsorption of MB, DOX-HCl, and BSA by PVA microspheres, at different times, are shown in Figure 9a. The amounts of the adsorbed MB and DOX-HCl increase rapidly during the first 60 min, and the maximum adsorption was observed at 2 h, which are 15.733 and 39.195 μg/mL, respectively. In contrast, only 3.626 μg/mL BSA is adsorbed. It suggests that PVA microspheres have good adsorption performance on small molecules, which could be used in hemodialysis for the adsorption of drugs. The desorption performance of PVA microspheres and the cycling times on MB are investigated in an ethanol solution for MB, which is an important agent dye and medicine, as shown in Figure 9b,c. The PVA microspheres could be recycled for more than 10 times and still maintain a good adsorption performance, which is of great importance for industrial applications. MB forms a monovalent organic cationic quaternary amine ionic group in aqueous solutions and deprotonate in ethanol solution, and the adsorption of MB on the PVA microspheres depends on the electrostatic and hydrogen-bonding interactions. At the beginning of the adsorption, the adsorption rate is fast, due to the presence of a large number of adsorption sites on the surface of PVA microspheres and the high concentration of MB. Then, the adsorption rate slows down and finally saturates when all available adsorption sites are fully occupied.

The adsorption behavior is analyzed using the pseudo-first-order, pseudo-second-order, and Weber’s intraparticle diffusion model [23]:

(9) $$\begin{array}{l} pseudo-first-order:\\ \;\frac{dq_{t}}{dt}=k_{1}(q_{e}-q_{t}) \end{array}$$

(10) $$\begin{array}{l} pseudo-second-order:\\ \;\frac{t}{q_{t}}=\frac{1}{k_{2}q_{e}{}^{2}}+\frac{t}{q_{e}}\; \end{array}$$

(11) $$\begin{array}{l} Weber’s\;intraparticle\;diffusion\;model:\\ \;q_{t}=k_{\mathrm{id}}t^{1/2}+c\; \end{array}$$

where *q*_t_ and *q*_e_ are the adsorption amounts at time (*t*) and at equilibrium (mg⋅g^−1^), respectively; *k*_1_, *k*_2_, and *k*_id_ are the pseudo-first-order rate constant (min^−1^), the pseudo-second-order rate constant (g⋅mg^−1^⋅min^−1^), and the internal diffusion rate constant (mg⋅g^−1^⋅min^−0.5^), respectively; *t* is the contact time (min); and *c* (mg g^−1^) represents the intercept related to the boundary layer thickness.

The regression plots of the pseudo-first-order kinetic model and pseudo-second-order kinetic model are shown in Figure S2a,b, respectively. The corresponding kinetic parameters for the two models are listed in Table 1. The linearity of the plot indicates the applicability of the two kinetic models. The correlation coefficient (*R*^2^) in the pseudo-second-order kinetic model was 0.99997, closer to 1.0 in comparison to the pseudo-first-order kinetic model (0.93073). Moreover, the calculated equilibrium adsorption capacity (*q*_e,cal_ = 15.785 μg⋅mL^−1^) with the pseudo-second-order kinetic model shows a good agreement with the experimental value of 15.733 μg⋅mL^−1^. Therefore, the pseudo-second-order model was more consistent with the adsorption process than the pseudo-first-order model.

The adsorption process of MB was studied thoroughly, with Weber’s intraparticle diffusion model. Figure S2c shows that two stages take place during the adsorption: a rapid adsorption stage and an adsorption equilibrium stage. It means that the intraparticle diffusion of MB from the surface to the inside of the PVA microspheres and the external film diffusion of MB from the solution to the surface of the PVA microspheres have the same rate, probably due to the numerous open-pore structures of microspheres, which provide a pathway for the rapid adsorption of MB on the PVA adsorption site. Thus, the adsorption and desorption of MB on the PVA microspheres are fast, and the PVA microspheres are recyclable.

## 4. Conclusions

We prepared monodispersed PVA microspheres, using on-chip droplet microfluidic technology in a highly controllable way. A PDMS chip consisting of a droplet generation, enhanced mixing, and pre-curing unit was constructed, and the process of preparing and controlling PVA microspheres on-chip was studied. The three functional units allowed us to precisely control the components inside droplets, the rapid and thorough mixing, and the pre-cure of the microspheres. The prepared microspheres had monodisperse size, good sphericity, uniform structure, and good adsorption performance. 

The pH, PVA/GA mole ratio, and flow rate are the key parameters affecting the fabrication of PVA microspheres on-chip, and thus influence the quality of PVA microspheres. PVA microspheres have fast and high adsorption capacity for methylene blue and doxorubicin hydrochloride, and can be used as drug and adsorbent carriers and for the removal of organic contaminants. The microfluidic platform is expected to be applicable to not only the continuous and rapid fabrication of uniform microspheres but also studying the mechanism of microchannel reaction, in combination with other online analytical tools.

## Figures and Tables

**Table 1:** Fitting kinetic parameter for the adsorption model.

Models	Model Parameters	*R* ^2^
Pseudo-first-order model	*q*_e,cal_ = 14.666 *k*_1_ = 0.0692 min^−1^	0.93073
Pseudo-second-order model	*q*_e,cal_ = 15.785 *k*_2_ = 0.014278 g·mg^−1^·min^−1^	0.99997
Internal diffusion model	*k*_id1_ = 2.62688 mg·g^−1^·min^−0.5^ *c*_1_ = 0.57408 *k*_id2_ = 0.00786 mg·g^−1^·min^−0.5^	0.98796

## Supplementary Materials

The following are available online at https://www.mdpi.com/1996-1944/12/22/3712/s1, Figure S1: The infrared spectrogram of pure PVA and synthesised PVA microspheres, Figure S2: (**a**) Pseudo-first-order; (**b**) Pseudo-second-order; (**c**) Internal diffusion models for the adsorption of MB onto PVA microspheres.
