# Supplementary material for: Microfluidic Rapid Fabrication of Tunable Polyvinyl Alcohol Microspheres for Adsorption Applications
Source: Materials (Basel). 2019 Nov 11;12(22):3712. doi: 10.3390/ma12223712 (PMC6888467; doi:10.3390/ma12223712)
Supplement: Supplementary file 1 [file materials-12-03712-s001.pdf]

materials

MDPI

Article

Microfluidic Rapid Fabrication of Tunable Polyvinyl Alcohol Microspheres for Adsorption Applications

Jianmei Wang [1,*], Xueying Wang [2,†], Pingan Zhu [3],Chengmin Chen [1], Jianchun Wang [1], Yan Li [1,*] and Liqiu Wang [3]

1 Energy Research Institute, Qilu University of Technology (Shandong Academy of Sciences), Jinan 250014, China; chencm@sderi.cn (C.C.); wangjc@sderi.cn (J.W.)
2 Key Laboratory of Interfacial Reaction & Sensing Analysis in Universities of Shandong, School of Chemistry and Chemical Engineering, University of Jinan, Jinan 250022, P. R. China; hm_wangxy@ujn.edu.cn
3 Department of Mechanical Engineering, The University of Hong Kong, Hong Kong Postcode, China; pazhu@hku.hk (P.Z.); chm_wangxy@ujn.edu.cn (L.W.)
† This author contributed equally to this paper.
* Correspondence: wangjm@sderi.cn (J.W.); liyan@sderi.cn (Y.L.)

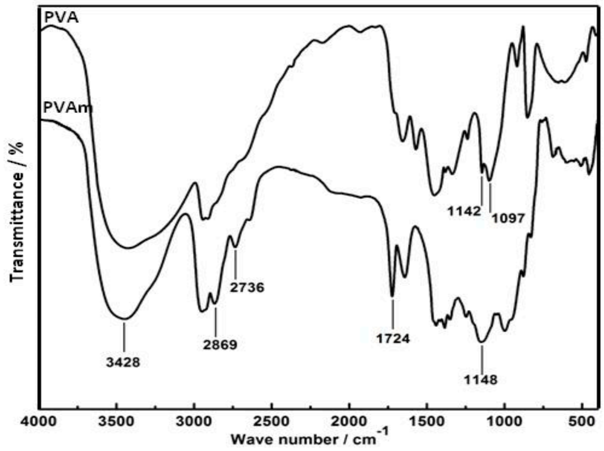

Figure 1. The infrared spectrogram of pure PVA and synthesised PVA microspheres.

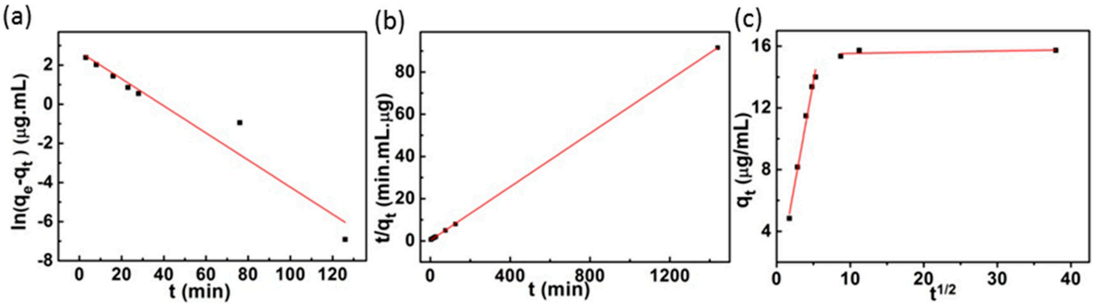

Figure 2. (**a**) Pseudo-first-order; (**b**) Pseudo-second-order; (**c**) Internal diffusion models for the adsorption of MB onto PVA microspheres.
